# Solid Tumor-Targeting Theranostic Polymer Nanoparticle in Nuclear Medicinal Fields

**DOI:** 10.1155/2014/424513

**Published:** 2014-10-14

**Authors:** Akira Makino, Shunsaku Kimura

**Affiliations:** ^1^Biomedical Imaging Research Center (BIRC), University of Fukui, Fukui 910-1193, Japan; ^2^Research and Education Program for Life Science, University of Fukui, Fukui 910-1193, Japan; ^3^Department of Material Chemistry, Graduate School of Engineering, Kyoto University, Kyoto 615-8510, Japan

## Abstract

Polymer nanoparticles can be prepared by self-assembling of amphiphilic polymers, and various types of molecular assemblies have been reported. In particular, in medicinal fields, utilization of these polymer nanoparticles as carriers for drug delivery system (DDS) has been actively tried, and some nanoparticulate drugs are currently under preclinical evaluations. A radionuclide is an unstable nucleus and decays with emission of radioactive rays, which can be utilized as a tracer in the diagnostic imaging systems of PET and SPECT and also in therapeutic purposes. Since polymer nanoparticles can encapsulate most of diagnostic and therapeutic agents with a proper design of amphiphilic polymers, they should be effective DDS carriers of radionuclides in the nuclear medicinal field. Indeed, nanoparticles have been recently attracting much attention as common platform carriers for diagnostic and therapeutic drugs and contribute to the development of nanotheranostics. In this paper, recent developments of solid tumor-targeting polymer nanoparticles in nuclear medicinal fields are reviewed.

## 1. Introduction

Nanoparticles have been actively examined as carriers for drug delivery system (DDS), which make it possible to improve therapeutic efficacy and to suppress side effects of the parent drug [1–5]. Among various types of nanoordered carriers including inorganic and lipid particles, polymeric micelles and vesicles, which are prepared from self-assembling process of amphiphilic polymers, can encapsulate in general hydrophobic and hydrophilic compounds in the hydrophobic core and inner aqueous cavity regions, respectively [6–10]. Further, not only surface modification but also functionalization of these nanoparticles is possible with ease by using suitably designed amphiphilic polymers. Therefore, polymeric nanoparticles have received much attention as the DDS carriers.

Additionally, nanoparticles are originally accumulated at the region where cells are rapidly proliferating, because leakage of nanoparticles can occur through the holes on immature blood vessels. Combined with the effect of undeveloped lymph system at tumor region, nanoparticles are retained there, leading to the passive accumulation. This phenomenon is called the enhanced permeability and retention (EPR) effect and is one of the most frequently utilized methodologies of nanoparticles for DDS [11–13]. On the basis of these reasons, polymeric nanoparticle is a suitable carrier for solid tumors-targeting DDS [8, 14]. In fact, various tumor-targeting DDS carriers have been developed, and some polymeric nanoparticles encapsulating antitumor drugs are currently under clinical trials [15].

Recently, polymeric nanoparticles are also applied as carriers for contrast agents in the field of* in vivo* imaging [16–18]. Among various modalities such as near-infrared fluorescence (NIRF) imaging, magnetic resonance imaging (MRI), positron emission tomography (PET), and single photon emission computed tomography (SPECT) systems, the* in vivo* near-infrared fluorescence (NIRF) imaging system has shown amazing progresses. This is because handling of the NIRF compounds is relatively easy and its biodistribution can be directly visualized. However, penetration depth of near-infrared light in tissue is limited to a few centimeters [19]. Therefore, the NIRF imaging system is suitable for* in vivo* experiments using small animals, but its applicative area in human clinical uses is limited to near the surface where fluorescent signal can be detectable. On the other hand, in general, sensitivity of nuclear imaging is high. Further, quantitative analyses are available by using PET images. When it comes to the quantitative performance, SPECT is less competitive than PET but has been improved by recent mechanical developments [20]. Positron emitters such as ^11^C, ^13^N, ^15^O, ^18^F, and ^64^Cu are used for PET imaging as signal sources, and *γ*-ray emitters such as ^99m^Tc, ^111^In⁡, and ^123^I are for SPECT imaging (Table 1). For preparation of nanoparticle type probes for nuclear imaging techniques, strategies on how to encapsulate these radioisotopes into nanoparticles are important. There are considerable approaches for the encapsulation, which can be classified into three categories: (1) hydrophobized radionuclides are encapsulated into the hydrophobic core region of polymeric micelle, (2) radionuclide solution is encapsulated into the inner cavity of vesicular assemblies, and (3) metal radionuclides such as ^64^Cu and ^99m^Tc can be attached to the nanoparticles as chelates by modifying constituent amphiphilic polymers with appropriate chelators. About the inner radiation therapy, *β* ^−^ ray emitters, such as ^90^Y and ^131^I, are generally used. In addition, attempts to utilize radionuclides emitting *α*-ray (^211^At, ^213^Bi) and auger electron (^99m^Tc, ^111^In⁡, and ^125^I) are also performed (Table 1) [21–25]. Any radionuclides labeled nanoparticle can be prepared by selecting appropriate methods for encapsulation as written above.

In this review, recent research developments on solid tumor-targeting DDS using polymer nanoparticle carriers in nuclear medicinal fields are summarized.

## 2. Polymeric Micelles

### 2.1. Importance of Surface Modification with Poly(ethylene glycol) (PEG)

#### 2.1.1. Poly(methyl acrylate)-*b*-poly(acrylic acid)

**Figure 1:**
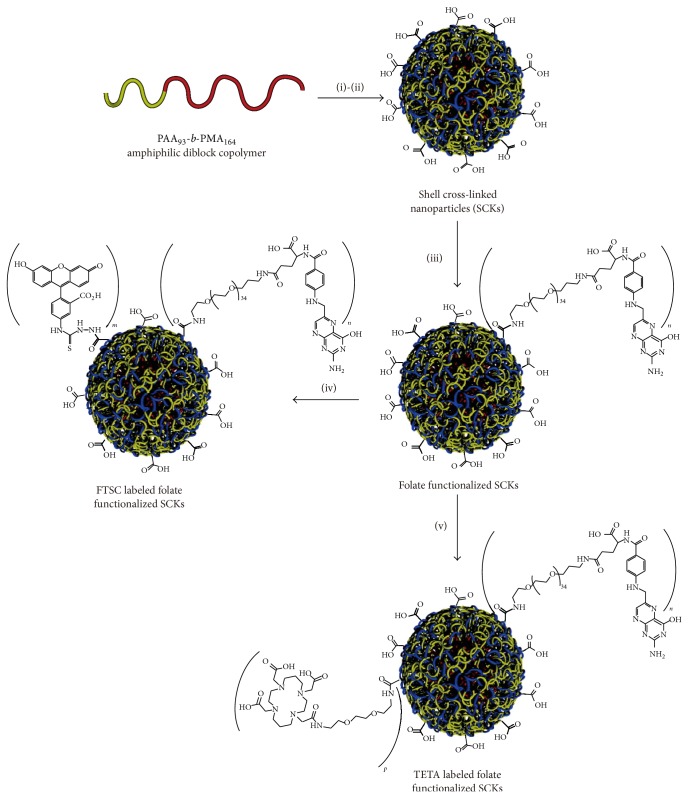
Polymeric micelle prepared from PMA-*b*-PAA. This research was originally published in JNM. R. Rossin, D. P. J. Pan, K. Qi, J. L. Turner, X. K. Sun, K. L. Wooley, and M. J. Welch. “Cu-64-labeled folate-conjugated shell cross-linked nanoparticles for tumor imaging and radiotherapy: Synthesis, radiolabeling, and biologic evaluation.” J. Nucl. Med. 2005, 46, 1210-1218. The Society of Nuclear Medicine and Molecular Imaging, Inc ©.

One of the initial trials to utilize polymeric micelle as a carrier for PET tracer agent was performed in 2005 by Rossin et al. [26]. Polymeric micelle was prepared from amphiphilic poly(methyl acrylate)-*b*-poly(acrylic acid) (PMA_164_-*b*-PAA_93_), which was synthesized via sequential atom transfer radical polymerization (ATRP) [27]. As illustrated in Figure 1, PMA-*b*-PAA in the micelle was cross-linked between the carboxyl acid group of PAA and the amine functionalities of 2,2′-(ethylenedioxy)diethylamine poly(acrylic acid) to form shell cross-linked (SCK) nanoparticles. Folate receptor (FR) is well-known molecular target for tumor therapies [28], and therefore, folate-poly(ethylene glycol)-amine was conjugated to the surface of the micelle. Further, 1,4,8,11-tetra-azacyclotetradecane-*N*′, *N*′′, *N*′′′, *N*′′′′-tetra-acetic acid (TETA) was also modified to the micelle surface for ^64^Cu chelate formation.

Using tumor transplanted mice, biodistribution of the micelle was examined by organ harvesting method. The micelle was recognized by reticuloendothelial system (RES), which is self-defense system of living organisms, and its undesired uptake at liver and spleen was high. However, owing to the combined effects of FR-mediated cell uptake and EPR effect, the micelle was accumulated at the tumor region with 2.1 ± 0.3, 3.2 ± 0.7, 6.0 ± 1.9, and 5.6 ± 0.9% ID/g at 10 min, 1 h, 4 h, and 24 h from the administration, respectively.

#### 2.1.2. Poly(methyl methacrylate-*co*-methacryloxysuccinimide-*graft*-poly(ethylene glycol))

**Figure 2:**
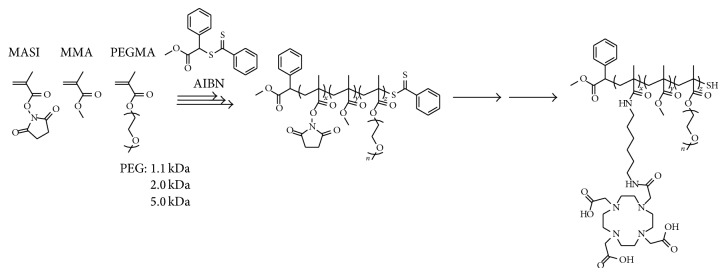
Scheme for poly(methyl methacrylate*-co*-methacryloxysuccinimide-*graft*-poly(ethylene glycol)).

To suppress undesired micelle uptake by RES, comb copolymers with branching various lengths of poly(ethylene glycol) (PEG) chains (1.1–5.0 kDa) were synthesized by standard reversible addition-fragmentation chain-transfer (RAFT) polymerization conditions [29], and to the polymer terminal end, 1,4,7,10-tetra-azacyclododecanetetra-acetic acid (DOTA) ligand was introduced as a chelator for radionuclides (Figure 2). The chelation with ^64^Cu was carried out after preparation of polymeric micelles. Diameters of the micelles prepared from copolymers with PEG of 1.1, 2.0, and 5.0 kDa were 9.7 ± 1.1, 17 ± 2, and 20 ± 3 nm, respectively.

Three types of ^64^Cu labeled micelles were injected into rats from the tail vein and their biodistribution was determined by organ harvesting method so as to evaluate the effect of PEG chain length on the micelle biodistribution. Additionally, the imaging studies were carried out using small animal PET system. The 5.0 kDa PEG micelle underwent a slow blood clearance, and 31 ± 2% of the dose was still in blood at 48 h after injection, which was almost ten times and twice higher than that of 1.1 kDa and 2.0 kDa PEG micelles, respectively. Biodistribution trend in the liver was opposite to that observed in the blood. Liver uptake of 1.1, 2.0, and 5.0 kDa PEG micelles at 24 h from the injection was decreased in this order and to be ca. 4.0, 2.8, and 1.2% ID/g, respectively. These results indicated that surface modification with PEG is important to control* in vivo* dynamics of the micelle, and prolongation of blood circulation time and low accumulation in excretory organs can be achieved by increased thickness of PEG shell.

#### 2.1.3. Poly(lauryl methacrylate)-*b*-poly(*N*-(2-hydroxypropyl)methacrylamide)

Amphiphilic copolymer consisted of hydrophobic poly(lauryl methacrylate) (poly(LMA)) and hydrophilic poly(*N*-(2-hydroxypropyl)methacrylamide) (poly(HPMA)) blocks that was synthesized via RAFT polymerization, and PEG_2000_ was incorporated into the side chain of the hydrophilic block with 0–11% modification ratio [30]. Further, the amphiphile was covalently labeled by radionuclides of ^18^F. Polymeric micelle was prepared from the amphiphile, and the effects of the PEGylation on micelle size, biodistribution, and cell uptake behavior were evaluated. In animal experiments using rats, polymeric micelle with diameter of 38.1 ± 2.1 nm, which was prepared from amphiphilic block copolymer with 7% PEGylation, exhibited the most favorable organ distribution pattern, showing highest blood circulation behavior as well as lowest undesired uptake at spleen and liver. Tumor cells of Walker 256 mammary carcinomas were clearly visualized by animal PET system after 2 h from the micelle dosage.

The group also synthesized random copolymers as well as block copolymers. Polymeric micelles were prepared from these copolymers, and their cellular uptake and biodistribution were evaluated using two different types of tumor models (AT1 prostate carcinoma and Walker-256 mammary carcinoma). They concluded that not only PEGylation, but also other numerous factors including molecular weight of the copolymers, polymer structure, size of the micelle, and characteristics of tumor affected the intratumor accumulation level of the micelle [31]. Therefore, preclinical screening to analyze polymer uptake for each individual patient is considered to be essential for each chemo- and radiotherapy using polymer-based DDS.

### 2.2. Core-Cross-Linked Type Polymeric Micelle (CCPM) for Enhanced Thermodynamic Stability

Compared with liposome, polymeric micelle is thermodynamically stable. This is because relatively long hydrophobic polymer chains can stabilize the molecular assemblies more than the hydrophobic interaction among alkyl chains of phospholipids. Aiming at further improvement of polymeric micelle stability, intermolecular cross-linking of copolymers via covalent bonds has been examined.

#### 2.2.1. Poly(triethoxysilyl propylmethacrylate)-*b*-poly(PEG-methacrylate)

**Figure 3:**
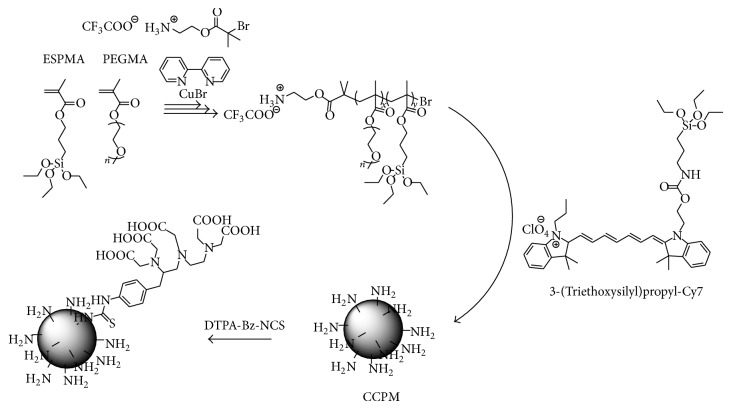
Preparation of core-cross-linked polymeric micelle (CCPM).

Poly(triethoxysilyl propylmethacrylate)-*b*-poly(PEG-methacrylate) (PESPMA-*b*-PPEGPMA) was synthesized by atom transfer radical polymerization (ATRP). From the mixture of the polymer and 3-(trimethoxysilyl)propyl-Cy7, polymeric micelle was prepared by sol-gel process upon addition of acetic acid. The acid caused rapid hydrolysis of ethoxy silane precursors and subsequent cross-link of the PESPMA core with the Cy7 derivative via Si–O bonds. Targeting the amino group, which is located at terminal end of hydrophilic block, DTPA was inserted on the surface of the core-cross-linked polymeric micelle (CCPM) and used for chelate formation with ^111^In⁡ (Figure 3). To the breast tumor cells of MDA-MB468 transplanted mice, ^111^In⁡-DTPA-CCPM with diameter of 24 ± 8.9 nm was injected and *γ*-scintigraphy and NIRF optical imaging were performed [32]. CCPM showed prolonged blood circulation behavior (*t*_1/2,*α*_ = 1.25 h, *t*_1/2,*β*_ = 46.18 h) and passively accumulated at the tumor region (5.5% ID/g at 48 h after injection). At 120 h from the dosage, tumor/blood and tumor/muscle signal ratios reached 4.44 and 28.0, respectively.

Characteristic point of CCPM is location of amino group on the surface, and therefore, it is possible to attach targeting ability to CCPM by ligand conjugation. For example, EphB4-binding peptide and synthetic somatostatin analogue of octreotide-conjugated CCPMs were prepared to detect EphB4-positive PC-3 M prostate and somatostatin receptor overexpressed glioblastoma U87 cells, respectively [33, 34]. When a cell undergoes apoptosis, phosphatidylserine is exposed on the cell surface. Then, annexin A5-conjugated CCPM, which binds strongly with phosphatidylserine, was also prepared [35]. Utilizing these ^111^In⁡ labeled CCPMs, dual SPECT and NIRF imaging of the targeted region was accomplished. Further, time for accumulation of these ligand-conjugated CCPMs to the targeted region could be shortened by specific bindings with corresponding receptors, and signal intensity ratio against background was also improved.

#### 2.2.2. Poly(7-(2-methacryloyloxyethoxy)4-methylcoumarin)-*b*-poly(hydroxyethylmethacrylate)-b-poly(ethylene glycol)

Copolymer consisting of one hydrophobic 7-(2-methacryloyloxyethoxy)-4-methylcoumarin (CMA) and two hydrophilic hydroxyethylmethacrylate (HEMA) and PEG blocks was also synthesized by Jensen et al. [36]. To hydroxyl groups of the HEMA block, DOTA or CB-TE2A was conjugated as a chelator for radioactive metal of ^64^Cu. After micelle formation by using self-assembling mechanism, the micelle dispersion was irradiated by UV light so as to cross-link 4-methylcoumarin moieties in the hydrophobic core. The cross-linked micelle showed higher stability in blood and was accumulated at transplanted tumor region (U87MG) of mice. After 22 and 46 h from the dosage of ^64^Cu labeled micelles, tumor can be visualized.

### 2.3. Micelle Formation from Biodegradable and Biocompatible Amphiphilic Polymers

#### 2.3.1. Poly(*ε*-caprolactone)-*b*-poly(ethylene glycol)

**Figure 4:**
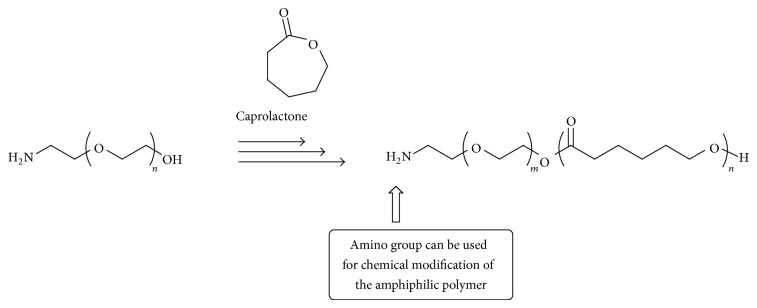
Amphiphilic poly(*ε*-caprolactone)-*b*-poly(ethylene glycol) for micelle preparation.

Polymeric micelle can be prepared from various amphiphilic polymers. From the view point that polymer nanoparticles are applied as carrier for DDS, utilization of biodegradable and biocompatible amphiphilic polymers should take priority. Then, poly(*ε*-caprolactone), which is representative biodegradable polymer connecting through ester linkages, was selected as hydrophobic block of the amphiphilic polymer (Figure 4) [37]. For the chelate formation with ^111^In⁡, terminal end of the amphiphilic polymer is modified by diethylene triamine penta-acetic acid (DTPA), which is a well-known chelator to form stable complexes with metals. From DTPA modified poly(*ε*-caprolactone)-*b*-poly(ethylene glycol) (PCL-*b*-PEG), polymer micelle was prepared and then radionuclide ^111^In⁡ was chelated to DTPA [38]. To the tumor-bearing mice, the ^111^In⁡ labeled micelle with diameter of ca. 60 nm was injected, and its biodistribution and pharmacokinetics were evaluated by the organ harvesting method and SPECT/CT* in vivo* imaging system. The micelle was passively accumulated at the tumor region (9 ± 2% ID/g), and transplanted tumor could be imaged by SPECT.

On a cell surface of various types of epithelial cancers including lung and breast, epidermal growth factor receptor (EGFR) is known to be overexpressed. The micelle surface was functionalized by conjugating EGF to terminal amino group of the amphiphile so as to improve the ^111^In⁡ labeled micelle delivering efficiency to the targeted tumor region [39]. Compared with the accumulation of the nontargeting micelle (EGF−),* in vivo* cell uptake and cell membrane binding of the EGF modified micelle (EGF+) to tumors overexpressing EGFR were significantly enhanced (*P* < 0.05).

^111^In⁡ can be also utilized as therapeutic nucleotide as emitter of auger electrons [40]. Using the EGF modified micelle, effect of auger electron therapy against EGFR-positive breast cancer cells was performed [41]. In this research, three types of human breast cancer cell cultures of MDA-MB-468 (1 × 10^6^ EGFR/cell), MDA-MB-231 (2 × 10^5^ EGFR/cell), and MCF-7 (1 × 10^4^ EGFR/cell) were used. Correlated with the density of EGFR expression level on these cancer cells, EGFR-mediated uptake of the micelle (EGF+) was increased. In* in vitro* cell survival assay, cell number of MDA-MB-468 was significantly decreased after 21 h from the treatment by 1 MBq of ^111^In⁡-DTPA modified micelle (EGF+). However, the micelle showed no cytotoxicity against MCF-7 with low EGFR expression level.

On the cell surface of breast cancer, it is known to express different levels of HER2. Then, ^111^In⁡ labeled micelle, whose surface is modified by HER2 specific antibody (trastuzumab fab) instead of the EGF sequence, was prepared, and its therapeutic performance was also evaluated [42, 43]. Targeting the gastric cancer diagnosis, glucose-regulated protein 78 (GRP78) binding peptide modified micelle was attached to the ^111^In labeled micelle surface, and* in vivo* SPECT imaging was accomplished [44]. In short, polymeric micelle can be arranged in accordance with the intended uses.

Preparation of multifunctional micelles for photothermal therapy (PTT) is also conducted [45]. To the hydrophobic core of the micelle prepared from DTPA modified PCL-*b*-PEG, near-infrared (NIR) dye of IR-780 iodide was encapsulated as signal agent for NIRF imaging and photosensitizer for PTT. Further, ^188^ *Re* was chelated to DTPA as a signal source for SPECT imaging. The micelle was designed so that* in vivo* dynamics after dosage can be traced by SPECT system. Based on the real time monitoring of the micelle integration to the targeted tumor region, NIR light irradiation could be carried out.

Characters of molecular assemblies can be controlled by changing polymer architecture and its hydrophilic-hydrophobic balance. For example, thermosensitive hydrogel containing a therapeutic radionuclide (^188^ *Re*-Tin colloid) and a chemotherapeutic drug (liposomal doxorubicin) was prepared from PCL-*b*-PEG-*b*-PCL triblock copolymer [46]. The thermosensitive gel was intratumorally administrated to the hepatocellular carcinoma, and the therapeutic effect was evaluated. At the tumor region, liposomal doxorubicin and ^188^ *Re*-Tin colloid were released slowly and steadily. The therapeutic effect was greater than the cases in which either component was individually used, and the synergetic effect against tumor growth was confirmed.

#### 2.3.2. Poly(ethylene glycol)-*b*-poly(lactic acid)

Poly(lactic acid) (PLA) is representative biodegradable polymer connecting through ester linkage. Polymeric micelle prepared from PEG-*b*-PLA is also evaluated and used as nanoordered carriers for DDS by various research groups [47].

**Figure 5:**
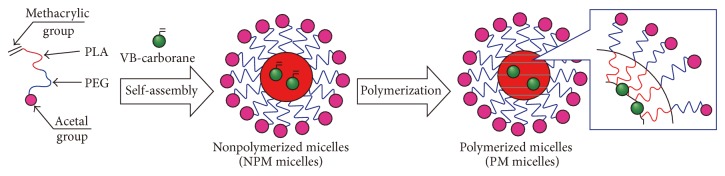
Schematic illustration of ^10^B-enriched micelle preparation from acetal-PEG-*b*-PLA-MA and polymerizable VB-carborane. Reprinted from Biomaterials, 2012, 33, 3568-3577. S. Sumitani, M. Oishi, T. Yaguchi, H. Murotani, Y. Horiguchi, M. Suzuki, K. Ono, H. Yanagie, and Y. Nagasaki. “Pharmacokinetics of core-polymerized, boron-conjugated micelles designed for boron neutron capture therapy for cancer.” Copyright (2012), with permission from Elsevier.

Nonradioactive ^10^B is known to produce *α* particles and ^7^Li nuclei with ca. 2.3 MeV of energy by the capture reaction of thermal neutrons, and therefore, utilization ^10^B on boron neutron capture therapy (BNCT) is investigated. BNCT could make it possible to irradiate directly targeted tumor region with suppressing nonspecific exposure. However, restricted neutron source for the treatment is one of the major problems for practical usages. Sumitani et al. evaluated utilization of PEG-*b*-PLA micelle to improve delivering efficiency of boron derivatives to the targeted tumor region [48, 49]. In this study, polymeric micelle was prepared from amphiphilic PEG-*b*-PLA polymer derivative of acetal-PEG-*b*-PLA-MA, whose PEG and PLA terminal ends are modified by acetal and methacryloyl groups, respectively. To the micelle, 1-(4-vinylbenzyl)-*closo*-carborane (VB-carborane) and azobisisobutyronitrile (AIBN) mixtures were encapsulated, and core cross-polymerized and boron-conjugated micelles were prepared by the free radical polymerization method (Figure 5). To the tumor transplanted mice, the ^10^B-enriched micelle was administrated, and neutron irradiation was performed after 24 h from the dosage. The ^10^B-enriched micelle was accumulated at the tumor region and showed significant therapeutic effects after the neutron irradiation.

#### 2.3.3. Poly(sarcosine)-*b*-poly(l-lactic acid)

Amphiphilic polydepsipeptide of (sarcosine)_70_-*b*-(l-lactic acid)_30_ is known to form polymeric micelle with diameter of ca. 35 nm, and the polymeric micelle was named as “Lactosome” [18, 50]. Sarcosine (Sar),* N*-methyl glycine in other words, is natural amino acid, and its homopolymer shows high solubility against aqueous solution like PEG. Therefore, polymeric micelle whose surface is covered with poly(Sar) was also expected to show a prolonged blood circulation behavior with low undesired accumulation by reticuloendothelial system (RES).

As a radionuclide for PET imaging, hydrophobized ^18^F by attaching PLLA chain ([^18^F]SFB labeled poly(l-lactic acid) of 30 mer) was encapsulated into the core region of the polymeric micelle by using hydrophobic interactions [51]. The ^18^F labeled Lactosome was administrated from the tail vein to the tumor transplanted mice, and PET images were taken after 6 h from the dosage. Lactosome showed a good blood circulation behavior owing to the surface modification with hydrophilic poly(sarcosine) chains. Therefore, signal intensity at organs with high blood flows was high. However, the accumulated signal in the transplanted tumor region could be detected. Since Lactosome surface was not specifically modified by ligands, Lactosome is considered to be passively accumulated to the tumor region by the EPR effect.

As a therapeutic radionuclide, [^131^I]SIB labeled PLLA was encapsulated into the Lactosome as *β* ^−^ ray emitter by the same approach with the previous ^18^F compound for PET imaging. To the tumor transplanted mice, which were preliminary treated with preethanol injection therapy (PEIT), ^131^I labeled Lactosome of 200 MBq/kg was injected and time courses of tumor growth were observed [52]. As a result, ^131^I labeled Lactosome could be delivered to the tumor region, and the tumor growth was significantly suppressed.

### 2.4. Accelerated Blood Clearance (ABC) Phenomenon of Lactosome

Lactosome is a candidate nanoordered carrier for drug and/or imaging agent delivery. On medicinal usages, nanocarriers are expected to show unaltered disposition on multiple administrations. However, production of anti-Lactosome antibody occurred after 3 days from the first Lactosome administration, and the antibody production kept high level for 6 months [53]. Lactosome on second dosage was opsonized by the anti-Lactosome antibody soon after the administration and entrapped by reticuloendothelial system (RES). This phenomenon is named as accelerated blood clearance (ABC) phenomenon of Lactosome, and resembled phenomenon is sometimes observed on PEGylated materials [54, 55].

**Figure 6:**
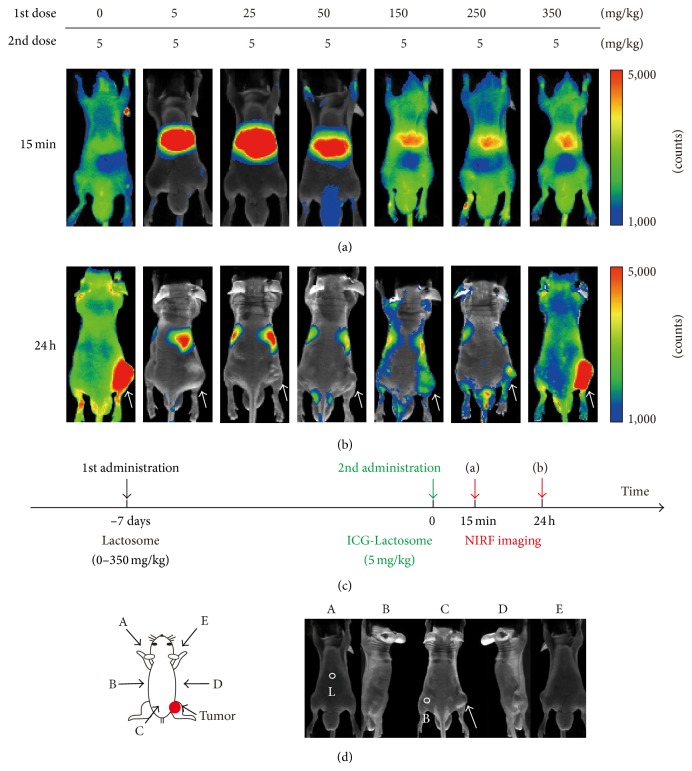
Effect of the first Lactosome dose on the Lactosome ABC phenomenon. NIRF images of mice at (a) 15 min (view from A in Figure 6(d)) and (b) 24 h (view from C in Figure 6(d)) after ICG-Lactosome of 2nd dose. Lactosomes (5, 25, and 50 mg/kg/100 *μ*L and 150, 250, and 350 mg/kg/200 *μ*L) were injected into the mice 7 days before the ICG-Lactosome administration. SUIT-2/pEF/luc cells were transplanted at the right femoral region of mice, in which tumor sites are indicated by white arrows. The fluorescein signal ranges were set to be the same for all the images from max count 5000 to min count 1000. (c) Time schedule for the NIRF imaging. Black and green arrows indicate the injection time points of Lactosome and ICG-Lactosome, respectively. NIRF imaging was performed at red arrows. (d) NIRF images were taken using Shimadzu Clairvivo OPT, which can take five images from different directions (A–E) with one time shot. The white circles indicate positions of ROI (L: liver; B: background). Reprinted from Biochemica et Biophysica Acta (BBA)-General Subjects, 2013, 1830, 4046–4052., E. Hara, A. Makino, K. Kurihara, M. Sugai, A. Shimizu, I. Hara, E. Ozeki, and S. Kimura. “Evasion from accelerated blood clearance of nanocarrier named as “Lactosome” induced by excessive administration of Lactosome.” Copyright (2013), with permission from Elsevier B.V.

The production amounts of the anti-Lactosome IgM were revealed to be inversely correlated with that of the first Lactosome dosage. When first Lactosome dosage is over 150 mg/kg, the Lactosome ABC phenomenon could be suppressed by induced immunological tolerance (Figure 6). Further, even if anti-Lactosome IgM is once produced, the Lactosome ABC phenomenon can be evaded by dosing Lactosome over 50 mg/kg. Importantly, acute toxicity was not observed at the Lactosome dosage amount [56].

Recently the production of anti-Lactosome IgM has been found to be suppressed by increasing the local density of poly(sarcosine) chains on the micelle surface. Higher local density of the surrounding hydrophilic polymer chains should be related with prevention of the interaction between micelles and B cell receptors [57].

Further, it is still unclear whether the ABC phenomenon is a general phenomenon for nanoparticles. However, to develop repeatedly injectable nanoordered carriers is essential for its general usages.

## 3. Vesicular Assemblies

The number of examples using vesicular assemblies as a carrier for DDS in nuclear medicinal field is relatively limited. It may be because of more difficult preparation of vesicular assemblies than polymeric micelles.

**Figure 7:**
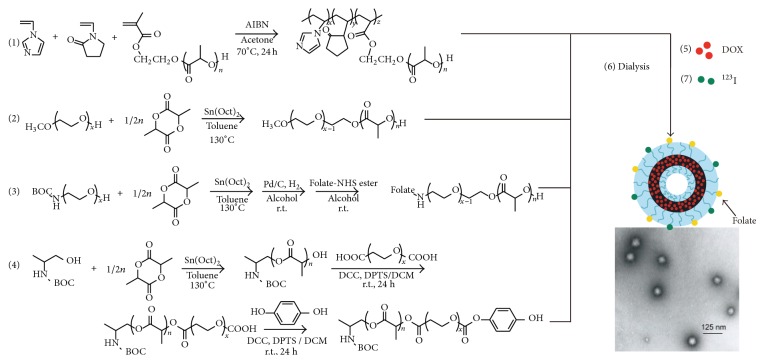
Representation of multifunctional hollow nanoparticles and their TEM image. Reprinted from Biomaterials, 2011, 32, 2213-2221., P. L. Lu, Y. C. Chen, T. W. Ou, H. H. Chen, H. C. Tsai, C. J. Wen, C. L. Lo, S. P. Wey, K. J. Lin, T. C. Yen, and G. H. Hsiue. “Multifunctional hollow nanoparticles based on graft-diblock copolymers for doxorubicin delivery.” Copyright (2011), with permission from Elsevier.

Lu et al. prepared multifunctional hollow nanoparticle from a mixture of doxorubicin hydrochloride (DOX-HCl), mPEG_5000_-*b*-poly(d,l-lactic acid)_1510_ (mPEG_5000_-*b*-PLA_1510_), folate-PEG_5000_-*b*-PLA_1200_, phenolic ester-PEG_5000_-*b*-PLA_650_, and poly(*N*-vinylimidazole-*co*-*N*-vinylpyrrolidone)_9600_-*g*-PLA_4900_ (Figure 7) [58]. The hollow nanoparticle is designed to show sensitivity against intracellular pH changes by using pH sensitive character of* N*-vinylimidazole (NVI) (pKa values of NVI are around 6.0). Depending on the pH changes of the dispersion, its diameter was reversibly changed between 90 nm (pH 7.4) and 80 nm (pH 5.0), and rate of encapsulated DOX-HCl release could be accelerated under weak acidic conditions. Extracellular matrix of the tumor region is known to be in a weakly acidic condition, and therefore, the DOX-HCl is expected to be released selectively at the tumor region. Folate-PEG_5000_-*b*-PLA_1200_ provided the nanoparticle with a tumor-targeting ability, and phenolic ester-PEG_5000_-*b*-PLA_650_ was used for modification of the nanoparticle with ^123^I by the iodogen method.

To the tumor transplanted mice, the multifunctional hollow nanoparticle was administrated, and the time course of tumor growth was observed. Dynamics of the nanoparticle at 0.5–6 h from the administration were noninvasively visualized by SPECT. Shortly after the administration, the nanoparticle was accumulated at the tumor region with a high level due to the folate-binding protein effect. Compared with the case of free DOX-HCl, the tumor growth was effectively suppressed with a minimum body weight loss.

## 4. Conclusion

In particular in these ten years, application of polymeric nanoparticles as a carrier for tumor-targeted drug delivery system (DDS) is expanded to nuclear medicinal fields. It is a tremendous advantage to apply polymeric nanoparticles for DDS, because* in vivo* pharmacokinetics can be well controlled by suitable selections about their size, shape, and surface characters and can be free from the loading imaging or therapeutic compounds. Therefore, when the same nanoparticle is used as a carrier for imaging and therapeutic purposes, diagnostic outcomes can directly predict the treatment efficiency of the loading therapeutic agent on the nanoparticle. Further, imaging results before and after chemotherapy can be used for prediction and evaluation of the therapeutic effect, respectively. In order to improve personal medical treatment, it is essential to dose the therapeutic agent properly to each patient. Polymeric micelles and vesicles endowed with two functions for diagnosis and therapy are expected to be potential materials for future personalized medicine, and these research areas are especially named as “nanotheranostics.”

However, there are many research problems to be dissolved. For example, almost all types of nanoparticles are designed to evade from undesired capture by reticuloendothelial system (RES) and to show a prolonged blood circulation behavior. Radioactivity is decreased with distinct half-life of each radionuclide, and time period, which can be effectively used, is limited. Therefore, radionuclides with relatively long half-life period such as ^64^Cu for PET and ^111^In⁡ for SPECT are mainly selected for radiolabeling of polymer nanoparticles. Further, how to control radiation exposure is also extremely important in nuclear medicinal fields. For these reasons, purpose of polymer nanoparticle DDS is mainly diagnosis, which can be performed by relatively low radioactivity, and research trials on inner radiotherapy are small in number. To accomplish practical usages of polymer nanoparticles as carriers for inner radiotherapy, (1) further improvement of drug delivery efficiency and (2) reduction of radiation exposure at radiosensitive organs such as bone marrow are essential by controlling the nanoparticles* in vivo* dynamics. It has already been revealed that to create synergetic therapeutic effect is possible by combined usages of therapeutic nanoparticle with conventional therapies, and therefore, therapeutic nanoparticles are now in an early phase of development.

Another problem for the view point of commercialization is how to ensure the quality of the nanoparticle. The number of examples utilizing polymeric nanoparticles as carriers for DDS in human clinical practice is limited. Therefore, to make rules for polymer nanoparticle production and their clinical studies is also considered to be important and started in government regulatory agencies [59].

There are many problems to be dissolved; however, application and commercialization researches on theranostic nanoparticles are expected surely to make future progresses in nuclear medicinal fields.

## Figures and Tables

**Table 1:** Representative radionuclides used for imaging and radiotherapy.

Radionuclide	Emission type	Half-life	Usage^1^
^ 11^C	**β** ^ +^ (99.8%), *γ* (0.2%)	20.39 min	I
^ 13^N	**β** ^ +^ (99.8%), *γ* (0.2%)	9.965 min	I
^ 15^O	**β** ^ +^ (99.9%), *γ* (0.1%)	2.037 min	I
^ 18^F	**β** ^ +^ (96.7%), *γ* (3.3%)	109.8 min	I
^ 32^P	**β** ^−^ (100%)	14.24 day	T
^ 64^Cu	**β** ^ +^ (17.4%), *γ* (43.6%),	12.70 h	T/I
	**β** ^−^ (39.0%)		
^ 89^Sr	**β** ^−^ (100%)	50.53 day	T
^ 90^Y	**β** ^−^ (100%)	64.00 h	T
^ 99m^Tc	**γ** (>99.9%)	6.015 h	T/I
^ 111^In	**γ** (100%)	2.805 day	T/I
^ 123^I	**γ** (100%)	13.22 h	I
^ 125^I	**γ** (100%)	59.40 day	T/I
^ 131^I	**β** ^−^	8.021 day	T/I
^ 186^Re	**β** ^−^, *γ*	3.718 day	T/I
^ 188^Re	**β** ^−^, *γ*	17.00 h	T
^ 211^At	**α**	7.214 h	T
^ 213^Bi	**α**	2.14 min	T
^ 225^Ac	**α**	10.0 day	T

^1^Radionuclide usage for imaging and therapy are abbreviated as “I” and “T,” respectively.
